# Linkage disequilibrium score regression identifies genetic correlations between hepatocellular carcinoma and clinically relevant traits

**DOI:** 10.1002/ijc.70136

**Published:** 2025-09-27

**Authors:** Younghun Han, Vikram R. Shaw, Jinyoung Byun, Aaron P. Thrift, Catherine Zhu, Donghui Li, Rikita I. Hatia, Robin Kate Kelley, Sean P. Cleary, Anna S. Lok, Paige M. Bracci, Jennifer B. Permuth, Roxana Bucur, Jennifer Knox, Jian‐Min Yuan, Amit G. Singal, Prasun K. Jalal, R. Mark Ghobrial, Yuko Kono, Dimpy P. Shah, Mindie H. Nguyen, Neehar D. Parikh, Richard Kim, Hui‐Chen Wu, Hashem El‐Serag, Ping Chang, Yun Shin Chun, Jian Gu, Chad Huff, Asif Rashid, Lu‐Yu Hwang, Alison P. Klein, Saira A. Khaderi, Ahmed O. Kaseb, Kathrine A. McGlynn, Lewis R. Roberts, Manal M. Hassan, Christopher I. Amos

**Affiliations:** ^1^ Institute for Clinical and Translational Research Baylor College of Medicine Houston Texas USA; ^2^ Comprehensive Cancer Center University of New Mexico Albuquerque New Mexico USA; ^3^ Department of Medicine Baylor College of Medicine Houston Texas USA; ^4^ Dan L Duncan Comprehensive Cancer Center Baylor College of Medicine Houston Texas USA; ^5^ Department of Gastrointestinal Medical Oncology The University of Texas MD Anderson Cancer Center Houston Texas USA; ^6^ Department of Epidemiology The University of Texas MD Anderson Cancer Center Houston Texas USA; ^7^ Helen Diller Family Comprehensive Cancer Center University of California San Francisco San Francisco California USA; ^8^ Division of Hepatobiliary & Pancreatic Surgery University of Toronto Toronto Ontario Canada; ^9^ Division of Gastroenterology and Hepatology, Department of Internal Medicine University of Michigan Ann Arbor Michigan USA; ^10^ Department of Epidemiology and Biostatistics University of California San Francisco San Francisco California USA; ^11^ Department of Gastrointestinal Oncology Moffitt Cancer Center Tampa Florida USA; ^12^ Department of Cancer Epidemiology Moffitt Cancer Center Tampa Florida USA; ^13^ University Health Network Princess Margaret Cancer Center and Toronto General Hospital Toronto Ontario Canada; ^14^ Cancer Epidemiology and Prevention Program, UPMC Hillman Cancer Center, Department of Epidemiology, Graduate School of Public Health University of Pittsburgh Pittsburgh Pennsylvania USA; ^15^ Division of Digestive and Liver Diseases The University of Texas Southwestern Medical Center Dallas Texas USA; ^16^ Department of Gastroenterology and Hepatology Baylor College of Medicine Houston Texas USA; ^17^ J.C. Walter Jr. Transplant Center Houston Methodist Hospital Houston Texas USA; ^18^ Division of Gastroenterology & Hepatology University of California San Diego San Diego California USA; ^19^ Mays Cancer Center The University of Texas Health Science Center San Antonio MD Anderson San Antonio Texas USA; ^20^ Division of Gastroenterology and Hepatology, Department of Epidemiology and Population Health Stanford University Medical Center Palo Alto California USA; ^21^ Department of Environmental Health Sciences, Mailman School of Public Health Columbia University New York City New York USA; ^22^ Division of Surgery, Department of Surgical Oncology The University of Texas MD Anderson Cancer Center Houston Texas USA; ^23^ Department of Pathology The University of Texas MD Anderson Cancer Center Houston Texas USA; ^24^ Department of Epidemiology, Human Genetics, and Environment Science The University of Texas Health Science Center at Houston Houston Texas USA; ^25^ Department of Oncology Sidney Kimmel Comprehensive Cancer Center at Johns Hopkins Baltimore Maryland USA; ^26^ Division of Abdominal Transplantation Baylor College of Medicine Houston Texas USA; ^27^ Division of Cancer Epidemiology and Genetics National Cancer Institute Rockville Maryland USA; ^28^ Division of Gastroenterology and Hepatology Mayo Clinic Rochester Minnesota USA

**Keywords:** genetic correlation, GWAS, hepatocellular carcinoma, heritability, UK Biobank

## Abstract

Hepatocellular carcinoma (HCC) mortality is increasing globally, partly due to the growing prevalence of nonviral liver diseases. Genome‐wide association studies (GWAS) have identified genetic variants associated with HCC development. Leveraging GWAS summary statistics and linkage disequilibrium score regression (LDSR), we investigated disease co‐development with hepatitis C virus‐negative (HCV‐negative) HCC to provide unique insights into HCC etiology and prioritize relationships for further causal inquiry. We utilized the LDSR statistical framework to estimate the genetic correlation and heritability between HCV‐negative HCC with 901 epidemiologic, behavioral, and clinical traits from the United Kingdom Biobank (UKBB). First, we set the threshold for observed scale heritability of each trait at 0.02 to ensure reliable inferences with adequate study power. Next, we observed significant positive genetic correlations between HCV‐negative HCC and blood‐based biomarkers of liver injury (ALT, GGT) and allostatic load (including glycated hemoglobin, blood pressure, and total albumin). We also identified a positive genetic correlation between HCV‐negative HCC and diseases associated with metabolic dysfunction‐associated steatotic liver disease (MASLD), including diabetes, hypertension, chronic ischemic heart disease, and others. Taken together, our results help to identify polygenic and pleiotropic signals related to different phenotypic traits associated with HCC and support further exploration of the predictive power of blood‐based biomarkers identified in this study for inferring HCC development among HCV‐negative individuals.

## What's New?

Hepatocellular carcinoma arises from a combination of environmental and genetic factors, with the latter playing a potentially more significant role in nonviral hepatocellular carcinoma. Here, the authors characterized the genetic correlations and heritability between nonviral hepatocellular carcinoma and 901 epidemiologic, behavioral, and clinical traits derived from the United Kingdom Biobank using linkage disequilibrium score regression. They found positive genetic correlations between nonviral hepatocellular carcinoma and blood‐based liver injury and allostatic load biomarkers as well as other diseases including diabetes, hypertension, and chronic ischemic heart disease. The findings offer an atlas of genetic associations for further investigation into hepatocellular carcinoma etiology.

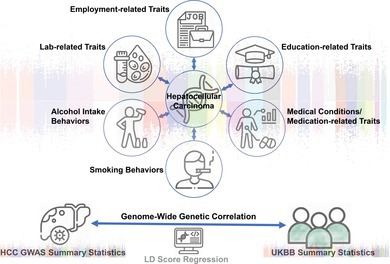

**Abbreviations**
ALD: alcohol‐associated liver disease
ALT: alanine aminotransferase
AST: aspartate aminotransferase
EHR: electronic health records
FDR: false discovery rate
GGT: gamma‐glutamyl transferase
GWAS: genome‐wide association studies
HBV: hepatitis B virus
HCC: hepatocellular carcinoma
HCV: hepatitis C virus
IBD: inflammatory bowel disease
LD: linkage disequilibrium
LDSR: linkage disequilibrium score regression
MAF: minor allele frequency
MASLD: metabolic dysfunction‐associated steatotic liver disease
MR: Mendelian randomization
SES: socioeconomic status
SNP: single‐nucleotide polymorphism
UKBB: United Kingdom Biobank

## 1 INTRODUCTION

Hepatocellular carcinoma (HCC) is a rising cause of cancer‐related death.^1^, ^2^ HCC pathogenesis demonstrates a strong environmental component, with heavy alcohol consumption, metabolic dysfunction‐associated steatotic liver disease (MASLD), hepatitis B virus (HBV), and hepatitis C virus (HCV) as the main risk factors.^2^ There has been a significant shift in the etiology of chronic liver disease, such that alcohol‐associated liver disease (ALD) and MASLD are now the leading causes of chronic liver disease and increasing causes of HCC.

Although environmental factors are a primary driver for HCC, family studies^3^, ^4^ and genome‐wide association studies (GWAS) have established a substantial genetic contribution to HCC development.^5^, ^6^, ^7^, ^8^ Our recent work focused on nonviral HCC among populations of European descent, identifying risk variants in *PNPLA3*, *HLA‐DQB1*, *TM6SF2*, *TERT*, *MAU2*, and *MOBP*.^8^ Additionally, mutational signature analyses of HCC tumors have identified dietary or medicinal exposure to aristolochic acid and cigarette smoking as cofactors that may influence HCC pathogenesis.^9^ The spectrum of mutations in HCC tumors is diverse; only about 25% of these tumors may contain actionable mutations, and the occurrence of individual mutations is generally low, indicating that most mutations are not prevalent across all HCC cases.^2^, ^9^, ^10^

HCC arises from a combination of environmental and genetic factors, with the latter playing a potentially more significant role in nonviral HCC,^8^ compared to viral HCC, and an opportunity exists to characterize this relationship further. Many environmental traits can be heritable or shared among individuals, although the extent and mechanisms can vary. For example, GWAS have identified risk variants associated with alcohol use in very large cohorts.^11^, ^12^ HCC development may be more likely in patients with both increased genetic risk for HCC and genetic risk for increased alcohol consumption, obesity, or type II diabetes mellitus.^13^ The first step towards evaluating that question is to characterize the shared genetic architecture between HCC risk and environmental, behavioral, and clinical traits, which may provide unique insights into shared genetic etiology in HCC and help to prioritize genetic and epidemiologic relationships for further causal inquiry.^14^, ^15^, ^16^, ^17^

Linkage disequilibrium (LD) score regression (LDSR) can be used to estimate the pairwise genetic correlation (*r*_*g*_) and heritability on the observed scale between two traits using GWAS summary statistics.^14^ By utilizing patterns of LD, LDSR regresses the product of single‐nucleotide polymorphism (SNP) *z*‐scores for a trait with a disease on the per‐SNP LD score to calculate the genetic correlation.^14^ Our previous work has used LDSR analysis to uncover shared genetic architectures within lung cancer,^17^, ^18^ covid‐19,^16^ primary sclerosing cholangitis,^15^ and inflammatory bowel disease (IBD),^19^ which highlighted unique shared associations, such as significant genetic correlations between multiple polygenic traits and diseases of interest.

In the present study, we characterized the genetic correlation and heritability on the observed scale (SNP‐heritability) between HCV‐negative HCC and 901 epidemiologic, behavioral, and clinical traits from the United Kingdom Biobank (UKBB) using LDSR, uncovering several genetic relationships among various pleiotropic and polygenic traits. We focused on HCV‐negative HCC as this was the primary group demonstrating significant SNP‐heritability.^8^ Our findings represent an atlas of interesting genetic associations that may prompt further investigation into HCC etiology.

## 2 MATERIALS AND METHODS

### 2.1 Summary statistics for HCC

The HCC summary statistics and detailed methodology for this study were previously published.^8^ In brief, a GWAS was performed using blood DNA from 1872 HCC patients and 2907 control individuals of European descent who had no evidence of cancer at the time of recruitment.^8^ Hepatitis B virus‐positive (HBV‐positive) patients were excluded from the original GWAS analysis since HBV is an uncommon cause of HCC in the United States.^8^ All participants were self‐reported non‐Hispanic whites.^8^ Genotyping was performed using the Illumina OmniExpressExome‐8v1‐3 platform with 958,497 SNPs, and ancestry inference analysis was performed to select individuals with ≥80% alignment of ancestral European descent.^8^, ^20^ After imputing GWAS data using the Haplotype Reference Consortium and following quality control procedures, association analysis was performed on ~12.6 million variants on autosomal chromosomes 1–22 and the X chromosome using logistic regression adjusted for age, gender, and the first 10 principal components.^8^ The final sample sizes used in the present study were 4779 individuals (*n* = 1872 cases and *n* = 2907 controls) for the overall analysis, 3453 individuals (*n* = 1134 cases and *n* = 2319 controls) for the HCV‐negative HCC subgroup, and 3645 individuals (*n* = 738 cases and *n* = 2907 controls) for the HCV‐positive HCC subgroup (Table 1).

**TABLE 1:** Heritability (h2_obs), standard error (SE), and sample size (*N*) for overall HCC, HCV‐negative (HCV−), and HCV‐positive (HCV+) strata.

Strata	h2_obs	SE	*N* (cases/controls)
Overall	−0.0246	0.0958	4779 (1872/2907)
HCV−	0.2022	0.1619	3453 (1134/2319)
HCV+	−0.0232	0.1102	3645 (738/2907)

*Note*: Negative heritability implies that the true value of h2 is low or the sample size is small.

### 2.2 Trait accession from the United Kingdom Biobank

Trait‐specific summary‐level data from the UKBB cohort were obtained for the following factors: alcohol behavior, blood and urine laboratory test results, body impedance measurement, diet, education and intelligence, employment, family history, lifestyle factors, medical conditions, medications, and smoking behavior. The UKBB is a prospective cohort study with data from over 500,000 participants aged 40–69 years recruited from 2006 to 2010 across the UK.^21^ The UKBB contains extensive phenotype and genotype data for participants, with 90% of the genotype data generated using a custom Affymetrix UKBB Axiom array.^21^ This array assayed ~850,000 SNPs, which were imputed through a collaboration with the Wellcome Trust Center for Human Genetics, resulting in a final count of 9.1 million SNPs.^21^ GWAS for each trait has been conducted on the imputed data, and summary statistics are publicly available (https://nealelab.github.io/UKBB_ldsc/).

### 2.3 Harmonization and quality control

Next, we performed data harmonization and additional quality control steps, following an approach previously published by us.^17^ For each chosen UKBB trait, SNP‐level effect sizes, *Z*‐scores, and standard errors are available. As a quality control measure, we filtered the UKBB imputed SNPs to those with a minor allele frequency (MAF) > 0.01, an imputation quality INFO score > 0.9, and removed SNPs not present in HapMap3, as previously described.^17^ A total of 1,073,880 SNPs were common between the HCC summary statistics and UKBB traits in the final LDSR analysis.

### 2.4 Linkage disequilibrium score regression analysis

The LDSR method has been previously described.^14^, ^17^ In brief, LDSR utilizes patterns of LD across the genome to calculate the shared heritability on the observed scale (h2_obs) and genome‐wide genetic correlation (*r*_*g*_) between traits.^14^ The genetic correlation is calculated by normalizing the genetic covariance by the calculated heritability of two compared traits, and the genetic covariance is obtained by regressing the product of SNP *z*‐scores by the per‐SNP LD score.^14^, ^17^ Notably, LDSR can be robust to cryptic relatedness and population stratification by including an intercept that accounts for genomic inflation.^17^, ^22^ However, this feature of LDSR was not necessary based on our results that showed no samples overlapped in pair‐wise kinship analyses of the HCC and the UKBB cohorts using PLINK options “‐‐make‐king‐table; ‐‐king‐table‐filter 0.0442 (third‐degree relationship).” We first estimated the heritability on the observed scale (h2_obs) for overall HCC and then separately for the HCV‐negative and HCV‐positive strata using LDSR (Table 1). Based on the LDSR package documentation, negative heritability implies that the true value of h2_obs is low or the sample size is small. Given that the overall HCC and HCV‐positive HCC groups have more samples than the HCV‐negative HCC group, the negative heritability likely arises from a true low SNP‐based heritability rather than an underpowered sample size. Next, we estimated genome‐wide pairwise genetic correlation (*r*_*g*_) of HCV‐negative HCC with 901 UKBB traits.

### 2.5 Statistical analysis

**FIGURE 1:**
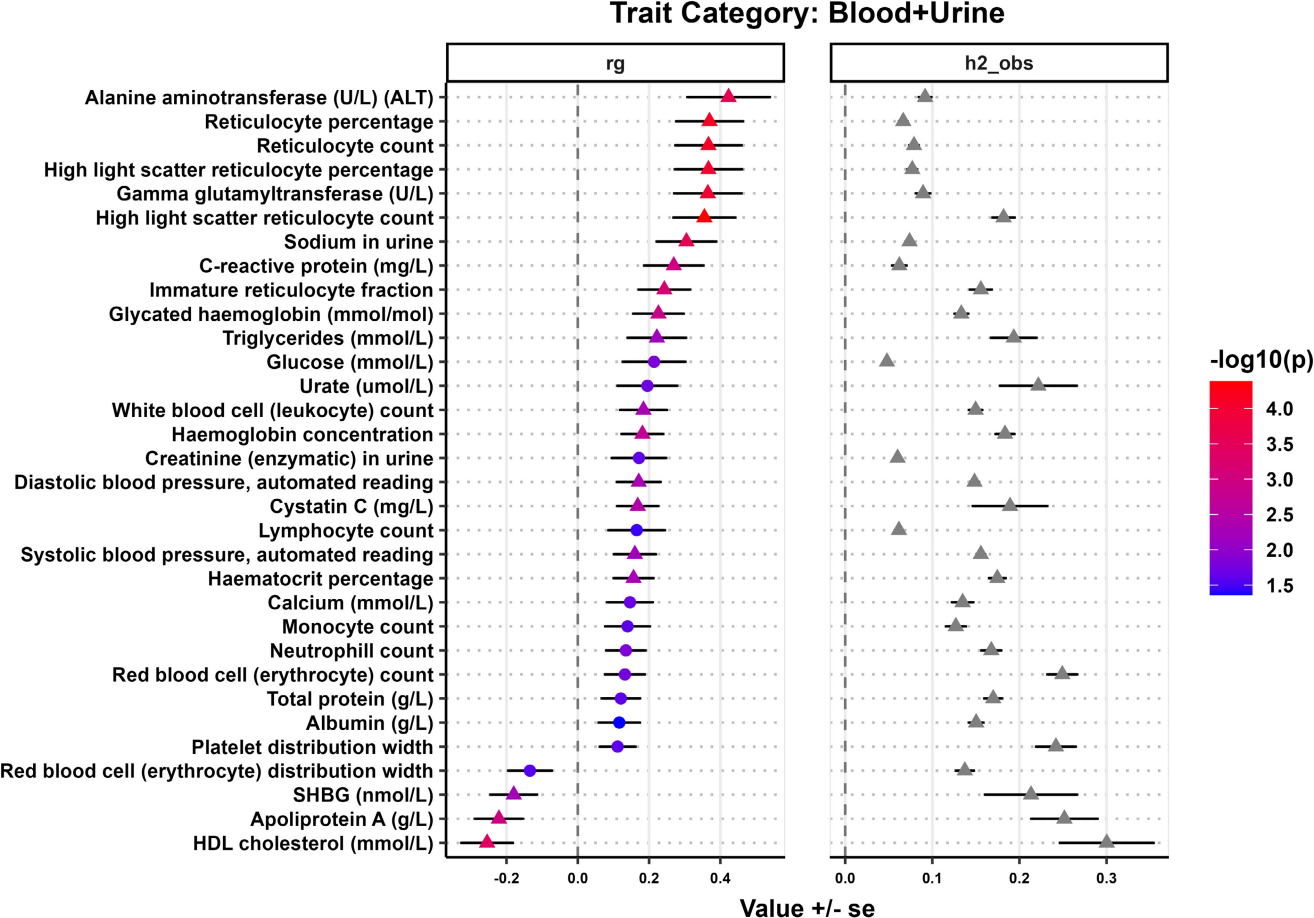
Genetic correlation (*r*_*g*_) and observed scale heritability (h2_obs) between blood and urine traits with HCV‐negative HCC. The circle indicates nominal significance (*p* ≤ 5 × 10^−2^), while the triangle indicates FDR‐adjusted significance (*p* ≤ 8.02 × 10^−3^).

**FIGURE 2:**
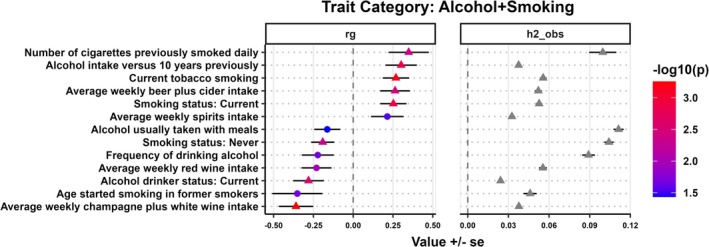
Genetic correlation (*r*_*g*_) and observed scale heritability (h2_obs) between alcohol and smoking traits with HCV‐negative HCC. The circle indicates nominal significance (*p* ≤ 5 × 10^−2^), while the triangle indicates FDR‐adjusted significance (*p* ≤ 8.02 × 10^−3^).

**FIGURE 3:**
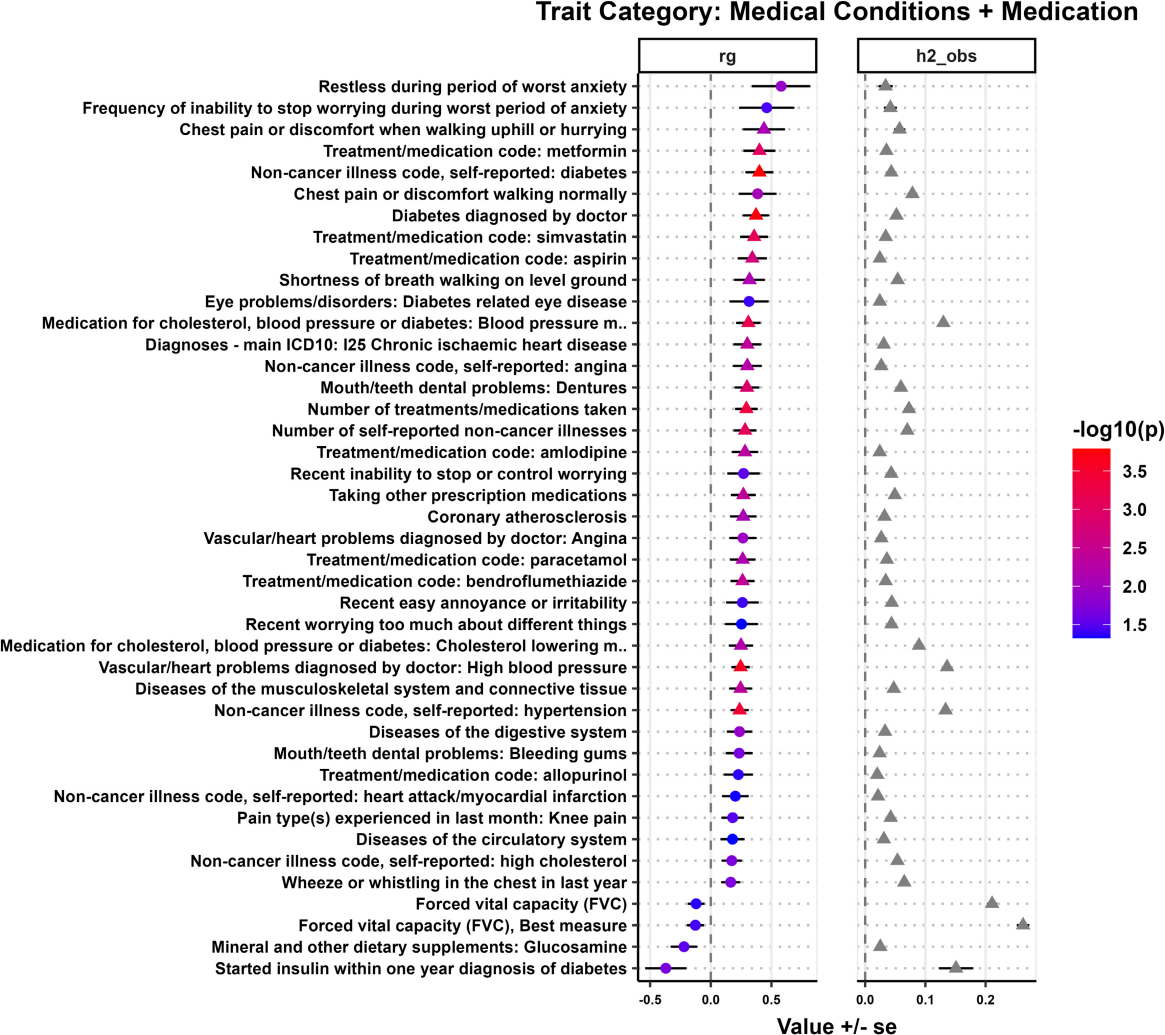
Genetic correlation (*r*_*g*_) and observed scale heritability (h2_obs) between medical conditions and medication traits with HCV‐negative HCC. The circle indicates nominal significance (*p* ≤ 5 × 10^−2^), while the triangle indicates FDR‐adjusted significance (*p* ≤ 8.02 × 10^−3^).

**FIGURE 4:**
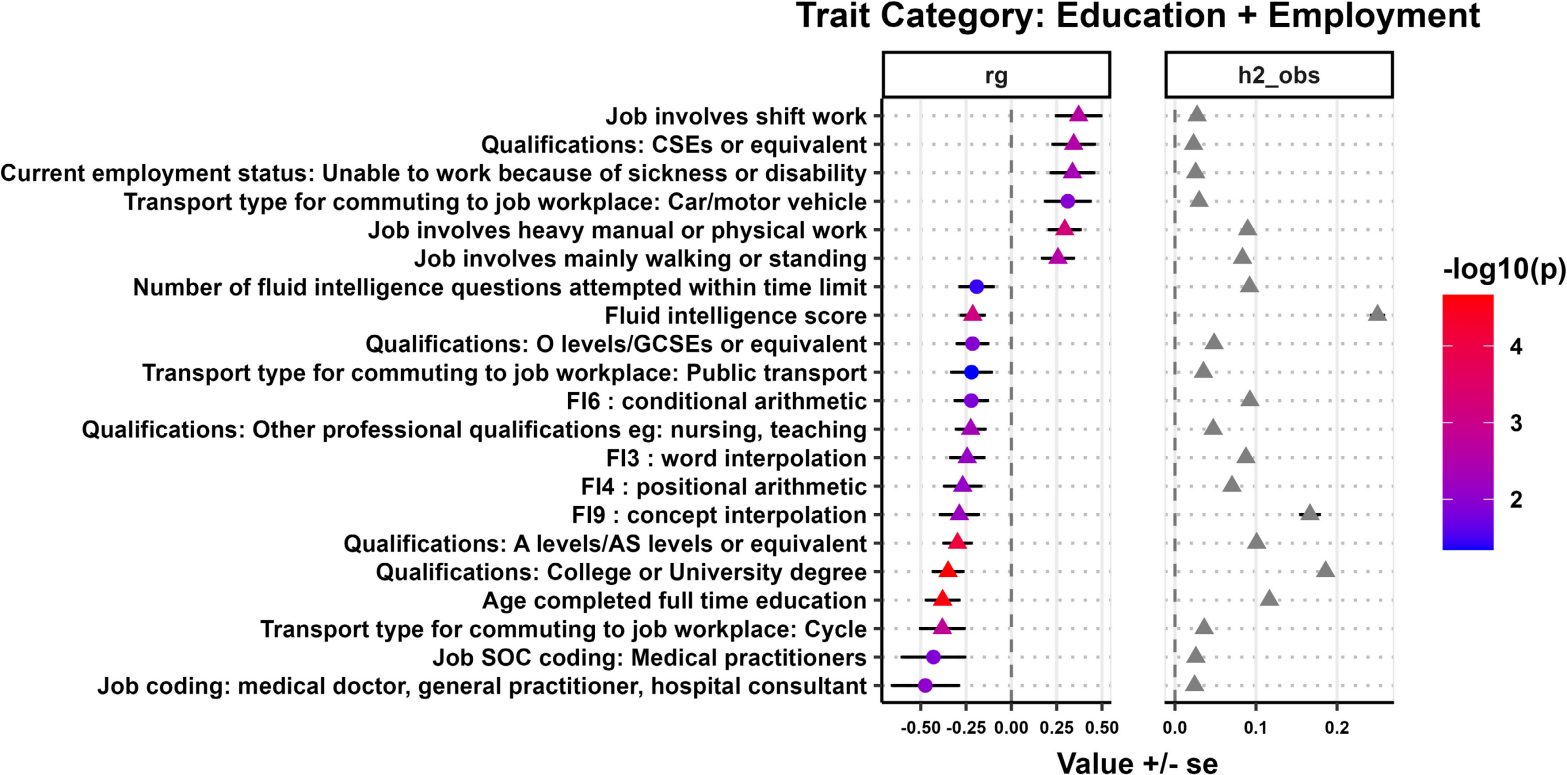
Genetic correlation (*r*_*g*_) and observed scale heritability (h2_obs) between education and employment traits with HCV‐negative HCC. The circle indicates nominal significance (*p* ≤ 5 × 10^−2^), while the triangle indicates FDR‐adjusted significance (*p* ≤ 8.02 × 10^−3^).

The 901 polygenic traits were first selected from an initial pool of 4228 UKBB traits by filtering for an h2_obs ≥0.02, indicating an observed scale heritability of at least 2% to retain reliable inferences with robust study power,^15^ and removing copies of duplicated traits and sex‐specific traits. Benjamini‐Hochberg false discovery rate (FDR) adjustment was then performed to account for multiple comparisons, including *r*_*g*_, h2_obs, and *p*‐values for each of the 901 pairwise comparisons. The *p*‐value corresponding to the 5% FDR (*p* ≤ 8.02 × 10^−3^) was used to establish the FDR‐adjusted statistical significance threshold, and comparisons were considered nominally significant at *p* ≤ 5 × 10^−2^. In Figures 1, 2, 3, 4 and S1–S5, Supporting Information, triangles were used to indicate traits meeting the FDR significance threshold, whereas circles were used to indicate traits meeting the nominal significance threshold.

### 2.6 Sensitivity analysis

To evaluate the robustness of our GWAS results and assess potential confounding from genetic loci associated with alcohol intake, we conducted a sensitivity analysis by excluding genomic regions previously implicated in alcohol consumption (⊥Alcohol). We identified genomic loci associated with alcohol intake from previously conducted GWAS.^12^ To exclude potential pleiotropic effects, we removed all SNPs located within ±500 kb of the lead SNPs reported in these loci from our GWAS summary statistics (Table S1). Genomic coordinates were based on the genome build GRCh37/hg19. After exclusion, we re‐ran the genetic correlation analysis using the LDSR. We compared the genetic correlation results between the primary and sensitivity analyses to assess whether the exclusion of alcohol‐associated regions materially impacted our findings. For the sensitivity analyses, we selected 64 traits with 5% FDR (*p* ≤ 8.02 × 10^−3^) among blood/urine, alcohol/smoking, medical conditions/medications, and education/employment traits.

## 3 RESULTS

### 3.1 HCC demonstrates differential observed‐scale heritability based on viral status

The observed‐scale heritability was −0.02 ± 0.10 for overall HCC, −0.02 ± 0.11 for HCV‐positive HCC, and 0.20 ± 0.16 for HCV‐negative HCC (Table 1). Based on the low heritability of HCV‐positive HCC and overall HCC, we did not further consider these subsets and restricted further analyses to HCV‐negative HCC.

### 3.2 Cross‐trait analysis between HCC and clinically relevant parameters revealed shared genetic architecture

We summarized the study design from data preparation to further downstream analyses in Figure S1. The 901 cross‐trait analysis outputs, including the genetic correlations (*r*_*g*_) and heritability (h2_obs), are available in Table S2. Additional figures for traits not included in the main results are available in Figures S2–S6.

### 3.3 Genetic correlations between blood and urine traits with HCV‐negative HCC

The pairwise genetic correlation was determined between blood and urine traits from the UKBB with HCV‐negative HCC (Figure 1). Alanine aminotransferase (ALT) and gamma‐glutamyl transferase (GGT) demonstrated strongly positive genetic correlations with HCV‐negative HCC (*r*_*g*_ = 0.42, *p* = 2.50 × 10^−4^; *r*_*g*_ = 0.36, *p* = 1.09 × 10^−4^, respectively). Additional positive correlations were seen between other common lab values with HCV‐negative HCC, including increased levels of glycated hemoglobin (*r*_*g*_ = 0.23, *p* = 1.37 × 10^−3^), hemoglobin concentration (*r*_*g*_ = 0.18, *p* = 1.89 × 10^−3^), glucose (*r*_*g*_ = 0.21, *p* = .015), reticulocyte count (*r*_*g*_ = 0.37, *p* = 7.73 × 10^−5^), urate (*r*_*g*_ = 0.20, *p* = .019), triglycerides (*r*_*g*_ = 0.22, *p* = 6.55 × 10^−3^), red blood cell (erythrocyte) count (*r*_*g*_ = 0.13, *p* = .018), and urine sodium (*r*_*g*_ = 0.30, *p* = 2.60 × 10^−4^). A nominally significant positive correlation was also observed between HCV‐negative HCC and immune cells, including the neutrophil, monocyte, and lymphocyte count. A negative genetic correlation was observed between HCV‐negative HCC with increased levels of HDL cholesterol (*r*_*g*_ = −0.25, *p* = 4.12 × 10^−4^) and apolipoprotein A (*r*_*g*_ = −0.22, *p* = 1.12 × 10^−3^).

### 3.4 Genetic correlations between alcohol intake and smoking behaviors with HCV‐negative HCC

Next, we analyzed the pairwise genetic correlations between HCV‐negative HCC with various traits related to smoking and alcohol use (Figure 2). A positive genetic correlation was observed between HCV‐negative HCC with average weekly beer plus cider intake (*r*_*g*_ = 0.26, *p* = 3.35 × 10^−3^) and average weekly spirits intake (*r*_*g*_ = 0.21, *p* = 2.93 × 10^−2^), while a negative genetic correlation was observed between HCV‐negative HCC and average weekly champagne plus white wine intake (*r*_*g*_ = −0.36, *p* = 5.55 × 10^−4^). A negative genetic correlation was observed between HCV‐negative HCC and current alcohol drinking status (*r*_*g*_ = −0.28, *p* = 2.56 × 10^−3^), but about 91.9% of the UK cohort population were surveyed as current drinkers based on “Alcohol drinker status (Data‐Field:20117).” A positive genetic correlation was observed between HCV‐negative HCC and current tobacco smoking (*r*_*g*_ = 0.27, *p* = 6.78 × 10^−4^) and the number of cigarettes previously smoked daily (*r*_*g*_ = 0.35, *p* = 4.36 × 10^−3^). A negative genetic correlation was observed between HCV‐negative HCC and no smoking history (*r*_*g*_ = −0.19, *p* = 4.93 × 10^−3^) and age started smoking in former smokers (*r*_*g*_ = −0.35, *p* = 2.20 × 10^−2^).

### 3.5 Genetic correlations between medical conditions and medications with HCV‐negative HCC

Next, we analyzed the pairwise genetic correlations between HCV‐negative HCC and various traits related to medical conditions and medications (Figure 3). A positive genetic correlation was observed between various physician‐diagnosed medical conditions or conditions present in patient electronic health records (EHR) and HCV‐negative HCC, including diabetes (*r*_*g*_ = 0.37, *p* = 1.81 × 10^−4^), hypertension (*r*_*g*_ = 0.25, *p* = 2.37 × 10^−4^), chronic ischemic heart disease (*r*_*g*_ = 0.30, *p* = 4.20 × 10^−3^), and angina (*r*_*g*_ = 0.27, *p* = 1.09 × 10^−2^). A positive genetic correlation was observed between HCV‐negative HCC and several specific medications, including metformin (*r*_*g*_ = 0.40, *p* = 1.06 × 10^−3^), simvastatin (*r*_*g*_ = 0.36, *p* = 8.06 × 10^−4^), aspirin (*r*_*g*_ = 0.32, *p* = 4.17 × 10^−3^), and amlodipine (*r*_*g*_ = 0.28, *p* = 5.00 × 10^−3^), in addition to general medication categories such as blood pressure and cholesterol‐lowering medications.

### 3.6 Genetic correlations between education and employment‐related traits with HCV‐negative HCC

Next, we analyzed the pairwise genetic correlations between HCV‐negative HCC and various traits related to education and employment (Figure 4). A negative genetic correlation was observed between HCV‐negative HCC and obtaining a college or university degree (*r*_*g*_ = −0.35, *p* = 2.18 × 10^−5^), professional qualifications (e.g., nursing or teaching) (*r*_*g*_ = −0.22, *p* = 4.75 × 10^−3^), fluid intelligence score (*r*_*g*_ = −0.21, *p* = 7.72 × 10^−4^), and age completed full‐time education (*r*_*g*_ = −0.38, *p* = 2.66 × 10^−5^). A positive genetic correlation was observed between HCV‐negative HCC and job‐related factors such as shift work (*r*_*g*_ = 0.37, *p* = 2.50 × 10^−3^), heavy manual or physical work (*r*_*g*_ = 0.29, *p* = 5.49 × 10^−4^), and jobs that mainly involve walking or standing (*r*_*g*_ = 0.26, *p* = 2.28 × 10^−3^).

### 3.7 Genetic correlations by excluding alcohol‐associated genomic loci

To attenuate the genetic contribution of alcohol consumption, we further conducted sensitivity analyses by pruning genomic regions associated with alcohol consumption and repeated LDSR analyses. After removing alcohol‐associated genomic loci, 1,041,896 SNPs remained for further LDSR analyses. We implemented a sensitivity analysis on 64 traits presented in Figures 1, 2, 3, 4. The results with or without including SNPs related to alcohol consumption were qualitatively similar. Pairwise genetic correlations between most traits studied with HCV‐negative HCC became more different from 0 as shown in Figures S7–S10, but the confidence intervals also increased, so that the actual p‐values of hypothesis tests of the correlation coefficients generally became less significant (Table S3).

## 4 DISCUSSION

The goal of the present study was to comprehensively examine the shared genetic correlation of HCV‐negative HCC with epidemiologic, behavioral, and clinical traits from the UKBB. We used LDSR,^17^, ^19^, ^22^ a novel approach to systematically estimate the genetic correlations between various traits and disease susceptibility, and uncovered relationships that provide improved polygenic and pleiotropic signals related to phenotypic traits associated with HCV‐negative HCC. Specifically, we validated expected results for HCV‐negative HCC, such as a positive genetic correlation with some patterns of alcohol use, and identified novel associations, such as positive genetic correlations with various blood and urine analytes.

First, our study investigated the pairwise genetic correlation between HCV‐negative HCC and various blood and urine traits. ALT and GGT demonstrated highly significant positive genetic correlations with HCV‐negative HCC. ALT and GGT are commonly used biomarkers for liver damage and disease.^23^ Accordingly, elevated ALT and GGT may be considered positive control results in the present study, which are expected to demonstrate a positive association with HCC. This suggests that other results, including the positive genetic correlations between HCV‐negative HCC and other blood/urine markers, warrant further inquiry, including reticulocyte count, urate, red blood cell count, glucose, triglyceride levels, and sodium in the urine. For example, lipidomics studies have suggested that changes in lipids, including triacylglycerols, may be involved in primary liver cancer development and progression, though further mechanistic work needs to be conducted.^24^ Lipidomic analyses were limited in this dataset. Although some blood and urine genetic correlations should be interpreted cautiously, particularly those with high daily or weekly variance (e.g., sodium in the urine), our findings support further assessment of relationships that underlie the genetic correlations between blood and urine markers with HCV‐negative HCC.

We identified many pairwise genetic correlations with blood biomarkers and medical conditions as components of allostatic load, broadly defined as the “cumulative burden of chronic stress and life events.”^25^ Chronic stress can affect cortisol, albumin, blood pressure, cholesterol, and glycated hemoglobin levels.^26^ In a model described by McEwen and McEwen, persistent stress may interact with genetic factors, precipitating metabolic changes that lead to disease development and progression.^26^ Allostatic load associated with HCC included increased blood pressure, glycated hemoglobin, albumin, cholesterol (via a positive genetic correlation with cholesterol‐lowering medications), decreased HDL cholesterol, and increased neutrophil count. This finding aligns with previously published research that demonstrated a positive correlation between an allostatic load score (which included measures of blood pressure, total serum cholesterol, and glycated hemoglobin) and the degree of hepatic steatosis and fibrosis in a multinomial logistic regression.^27^ Although research into allostatic load is a nascent field in liver pathophysiology and HCC, our results showing high genetic correlations between many allostatic load contributors and nonviral HCC support continued investigation of this relationship.

Overall, our results provide additional data for the role of alcohol consumption and tobacco smoking in HCC. A strong positive correlation was noted for overall patterns of intake of all types of alcohol. Interestingly, however, genetic correlation differences were observed when looking at the specific types of alcohol, and this relationship may be partly confounded by the effect of socioeconomic status (SES) on the type of alcohol consumed.^28^, ^29^ For example, a positive genetic correlation was observed between the increased average weekly beer plus cider intake and average weekly spirits intake with HCV‐negative HCC, while a negative genetic correlation was observed for the increased number of weekly champagne drinks plus white wine intake and average weekly red wine intake. This trend may be explained by a correlation between higher SES and wine consumption, while lower SES is correlated with beer consumption. Our study is limited to genetic correlation and is not a causal analysis, so confounding with SES could explain some of the variability in effects from sources of alcohol exposure.^28^, ^29^ Further studies that dissect complex patterns of risk associated with patterns of alcohol consumption are needed to analyze this relationship further. A recent analysis utilizing Mendelian randomization (MR), which aims to estimate the genetically predicted causal effect of an exposure trait on an outcome trait, identified a prominent J‐shaped risk pattern for wine drinking with decreased risk for light drinking and increased risk for abstinence or high consumption.^30^ Liu et al. also found that moderate wine drinkers had significantly lower alanine transaminase (ALT) and aspartate aminotransferase (AST) levels than non‐drinkers.^30^ In our analyses, there is an increased risk for HCC according to beer consumption, whiskey or other spirit consumption, and negative correlations for wine or regular alcohol use. These findings indicate a complex relationship between alcohol use and HCC risk that has previously been studied in participants in the UKBB.^30^ The observed negative genetic correlation between HCV‐negative HCC and current alcohol drinking status is surprising, given the known hepatocarcinogenic effects of alcohol. A limitation of the UKBB alcohol phenotype is that it does not distinguish lifelong abstainers from former drinkers. As a result, the “non‐drinker” category likely includes individuals who abstain due to prior heavy use or underlying health conditions, factors associated with increased HCC risk. This variable pattern of alcohol consumption could introduce collider bias or residual confounding with the current status of alcohol consumption, leading to a spurious inverse genetic correlation. In our analysis, we found that specific patterns of alcohol consumption and, in particular, beer and spirit consumption, increase HCV‐negative HCC risk.

The contribution of alcohol intake to HCC risk remains incompletely understood. While most epidemiological studies report an increased risk of HCC among heavy alcohol consumers, some have paradoxically observed a potential lower risk association with moderate wine consumption.^28^ To validate whether the observed genetic association of HCC with 64 pleiotropic and polygenic traits is truly due to direct biological effects or confounded by genetically driven alcohol behavior, we implemented a sensitivity analysis by excluding the alcohol‐associated loci. Stronger genetic correlation after exclusion of alcohol‐associated loci may suggest that alcohol‐associated loci may introduce pleiotropy, masking true genetic correlation. In addition, excluding these loci may help us uncover stronger correlations with traits that share a more direct genetic basis with HCC, and alcohol‐related loci might suppress the signal from other biologically meaningful genes by introducing variance unrelated to the core HCC mechanisms. Thus, genetic correlation before excluding these loci reflects a mixture of direct and behaviorally mediated effects, with possible pleiotropic distortion, and after excluding these loci, we can observe a stronger shared etiology of HCC among blood/urine, alcohol/smoking, medical conditions/medications, and education/employment traits.

Next, we investigated the pairwise genetic correlations between HCV‐negative HCC with medical conditions and medications. The positive genetic correlations among HCV‐negative HCC with diabetes, hypertension, chronic ischemic heart disease, and angina are consistent with nonviral HCC pathogenesis that may arise from metabolic dysfunction‐associated steatotic liver disease (MASLD).^31^, ^32^ The positive genetic correlation with medications for treating metabolic syndrome (e.g., metformin, simvastatin, aspirin, amlodipine) further supports the associations with medical history. Previous GWAS and candidate gene studies have improved the genetic understanding of MASLD, such as the I148M *PNPLA3* variant or variants in *TM6SF2*, *MBOAT7*, and *GCKR*.^33^ The results from this study suggest that patients with MASLD and HCC may have an underlying shared genetic architecture. Future work is needed to validate these shared genomic regions and potentially shared variants.

Based on results from our previous work in lung cancer^17^ and IBD,^19^ we examined the pairwise genetic correlations between HCV‐negative HCC and education‐related traits. Consistent with associations identified in our studies of these other diseases, we observed a negative genetic correlation between HCV‐negative HCC and educational attainment, such as obtaining a college or university degree (*r*_*g*_ = −0.35, *p* = 2.18 × 10^−5^), professional qualifications (e.g., nursing or teaching) (*r*_*g*_ = −0.22, *p* = 4.75 × 10^−3^), and the fluid intelligence score (*r*_*g*_ = −0.21, *p* = 7.72 × 10^−4^). These results may reflect the impact of education on HCC, potentially mediated by both SES (e.g., through access to care) and behavioral decision‐making (e.g., healthier lifestyle choices), and are consistent with previous studies showing that low SES is associated with increased HCC incidence.^34^

The present study has several limitations. First, LDSR analysis is useful for establishing the genetic correlation among traits, but it does not estimate the causal effect of an exposure trait on an outcome trait. MR analysis is a future step that may elucidate causal relationships between exposure traits and HCC as an outcome trait. While MR has strengths as a causal modeling tool, it also requires the selection of specific genomic instruments and has assumptions that require additional research. Successful application of MR requires that large samples are available for both the traits of interest and the disease outcomes. The UK Biobank provides large samples for the traits, but additional larger studies would be helpful to support future MR analyses. Second, LDSR is not likely to account for confounders, in which separate variable(s) may affect the exposure and the outcome traits, or mediators, in which separate variable(s) lies on the causal path from the exposure to the outcome. Third, as previously mentioned, blood and urine traits with high daily or weekly variance should be interpreted cautiously and require validation in independent studies. Finally, the present study focuses primarily on patients of European descent, thereby restricting inferences to other racial and ethnic groups; future studies are needed among diverse populations when the summary‐level GWAS data become available.

To summarize, the present study provides an atlas of cross‐trait genetic correlations in HCV‐negative HCC and provides new genetic confirmation of epidemiologic associations that have been observed in HCV‐negative HCC. Despite the limited sample size available for the initial analysis of HCC, we observed a surprisingly deep and extensive output of relevant biomarkers and environmental factors associated with the genetic architecture of HCV‐negative HCC development. LDSR is a robust statistical tool to identify polygenic and pleiotropic associations related to different phenotypic traits associated with HCV‐negative HCC susceptibility, and the present hypothesis‐generating study may serve as a starting point for future hypothesis‐driven work. In particular, the blood‐based biomarkers we identified showing genetic correlations with HCC risk should be further studied to understand the underlying mechanisms better and validate their role as markers for the future development of HCC among HCV‐negative individuals.

## AUTHOR CONTRIBUTIONS

**Younghun Han:** Conceptualization; methodology; data curation; formal analysis; supervision; investigation; writing – review and editing; software; resources; funding acquisition. **Vikram R. Shaw:** Writing – original draft; visualization. **Jinyoung Byun:** Conceptualization; writing – review and editing; supervision; investigation. **Aaron P. Thrift:** Writing – review and editing; funding acquisition; investigation. **Catherine Zhu:** Writing – review and editing. **Donghui Li:** Writing – review and editing. **Rikita I. Hatia:** Writing – review and editing. **Robin Kate Kelley:** Writing – review and editing. **Sean P. Cleary:** Writing – review and editing. **Anna S. Lok:** Writing – review and editing. **Paige M. Bracci:** Writing – review and editing. **Jennifer B. Permuth:** Writing – review and editing. **Roxana Bucur:** Writing – review and editing. **Jennifer Knox:** Writing – review and editing. **Jian‐Min Yuan:** Writing – review and editing. **Amit G. Singal:** Writing – review and editing. **Prasun K. Jalal:** Writing – review and editing. **R. Mark Ghobrial:** Writing – review and editing. **Yuko Kono:** Writing – review and editing. **Dimpy P. Shah:** Writing – review and editing. **Mindie H. Nguyen:** Writing – review and editing. **Neehar D. Parikh:** Writing – review and editing. **Richard Kim:** Writing – review and editing. **Hui‐Chen Wu:** Writing – review and editing. **Hashem El‐Serag:** Writing – review and editing. **Ping Chang:** Writing – review and editing. **Yun Shin Chun:** Writing – review and editing. **Jian Gu:** Writing – review and editing. **Chad Huff:** Writing – review and editing. **Asif Rashid:** Writing – review and editing. **Lu‐Yu Hwang:** Writing – review and editing. **Alison P. Klein:** Writing – review and editing. **Saira A. Khaderi:** Writing – review and editing. **Ahmed O. Kaseb:** Writing – review and editing. **Kathrine A. McGlynn:** Writing – review and editing; investigation. **Lewis R. Roberts:** Investigation; funding acquisition; writing – review and editing. **Manal M. Hassan:** Investigation; funding acquisition; writing – review and editing. **Christopher I. Amos:** Conceptualization; methodology; investigation; supervision; funding acquisition; writing – review and editing.

## CONFLICT OF INTEREST STATEMENT

R.M.G. has served as a consultant for Sanofi, received an honorarium from Sanofi for lectures, participated on the Advisory Board for Sirtex, TransMedics (not currently an Advisory Board Member), and LyGenesis, held leadership roles in the International Liver Transplantation Society and the American Society of Transplant Surgeons, owned but sold all stocks in TransMedics and Eli Lilly. M.H.N. received grants from Pfizer, Astra Zeneca, Glycotest, GSK, Delfi, Innogen, Exact Science, CurveBio, Gilead, Science, CurveBio, Gilead, Helio Health, National Institutes of Health, and Roche. She had been a consultant or on the advisory board of GSK and Exelixis. L.R.R. received grants from Boston Scientific, Exact Sciences, FUJIFILM Medical Systems, Gilead Sciences, Glycotest, Innovo Bioanalysis, Numares, Prometrika‐Surface Oncology, and TARGET PharmaSolutions. Speaker for Answers in CME, Roche, and Science First Communications. Advisory boards for AlphaSight, Novartis Venture Fund, AstraZeneca, Exact Sciences, and Gilead Sciences. The remaining authors have no conflicts to report.

## ETHICS STATEMENT

All participants provided informed consent according to protocols evaluated by the local Ethics Committee/Institutional Review Boards of the contributing study centers. Ethics approval for the UKBB was provided by the UK National Health Service (NHS) Research Ethics Service North West (Research Ethics Committee approval number 11/NW/0382), and all participants informed written consent. All methods were performed in accordance with the ethical guidelines of the 1975 Declaration of Helsinki.

## Supporting information


**Data S1.** Supporting Information.

**Data S2.** Supporting Information.

## Data Availability

The dataset supporting the conclusions of this manuscript is publicly available in Neale's lab repository for the UKBB GWAS summary statistics (https://nealelab.github.io/UKBB_ldsc/). Genotype and clinical data from the HCC cohort (except HCV status) are available in dbGaP under accession phs001744.v1.p1 upon Data Access Request approval. Further information is available from the corresponding author upon request.
