# Supplementary material for: Linkage disequilibrium score regression identifies genetic correlations between hepatocellular carcinoma and clinically relevant traits
Source: Int J Cancer. 2025 Sep 27;158(5):1193–203. doi: 10.1002/ijc.70136 (PMC12765977; doi:10.1002/ijc.70136)
Supplement: Supplementary file 2 — Data S2. Supporting Information. [file IJC-158-1193-s001.pdf]

Supplementary Information

Linkage disequilibrium score regression identifies genetic correlations between hepatocellular carcinoma and clinically relevant traits.

Younghun Han, Vikram Shaw, Jinyoung Byun, Aaron P. Thrift, Catherine Zhu, Donghui Li, Rikita I. Hatia, Robin Kate Kelley, Sean P. Cleary, Anna S. Lok, Paige M. Bracci, Jennifer B. Permuth, Roxana Bucur, Jennifer Knox, Jian-Min Yuan, Amit G. Singal, Prasun K. Jalal, R. Mark Ghobrial, Yuko Kono, Dimpy P. Shah, Mindie H. Nguyen, Neehar D. Parikh, Richard Kim, Hui-Chen Wu, Hashem El-Serag, Ping Chang, Yun Shin Chun, Jian Gu, Chad Huff, Asif Rashid, Lu-Yu Hwang, Alison P. Klein, Saira A. Khaderi, Ahmed O. Kaseb, Kathrine A. McGlynn, Lewis R. Roberts, Manal M. Hassan, Christopher I. Amos

Table of Contents

Supplementary Figure 1. Study design schematic for identifying the shared genetic etiology underlying HCC.

Supplementary Figure 2. Genetic correlation (r_g) and observed scale heritability (h2_obs) between body measurement impedance with HCV-negative HCC. The circle indicates nominal significance ($p \leq 5\times10^{-2}$), while the triangle indicates FDR-adjusted significance ($p \leq 8.02\times10^{-3}$).

Supplementary Figure 3. Genetic correlation (r_g) and observed scale heritability (h2_obs) between diet-related traits with HCV-negative HCC. The circle indicates nominal significance ($p \leq 5\times10^{-2}$), while the triangle indicates FDR-adjusted significance ($p \leq 8.02\times10^{-3}$).

Supplementary Figure 4. Genetic correlation (r_g) and observed scale heritability (h2_obs) between family history traits with HCV-negative HCC. The circle indicates nominal significance ($p \leq 5\times10^{-2}$), while the triangle indicates FDR-adjusted significance ($p \leq 8.02\times10^{-3}$).

Supplementary Figure 5. Genetic correlation (r_g) and observed scale heritability (h2_obs) between traits related to various life factors with HCV-negative HCC. The circle indicates nominal significance ($p \leq 5\times10^{-2}$), while the triangle indicates FDR-adjusted significance ($p \leq 8.02\times10^{-3}$).

Supplementary Figure 6. Genetic correlation (r_g) and observed scale heritability (h2_obs) between other traits with HCV-negative HCC. The circle indicates nominal significance ($p \leq 5\times10^{-2}$), while the triangle indicates FDR-adjusted significance ($p \leq 8.02\times10^{-3}$).

Supplementary Figure 7. Pairwise genetic correlation (r_g) between blood and urine traits with HCV-negative HCC on inclusion and exclusion of alcohol-associated loci.

Supplementary Figure 8. Pairwise genetic correlation (r_g) between alcohol and smoking traits with HCV-negative HCC on inclusion and exclusion of alcohol-associated loci.

Supplementary Figure 9. Pairwise genetic correlation (r_g) between medical conditions and medication traits with HCV-negative HCC on inclusion and exclusion of alcohol-associated loci.

Supplementary Figure 10. Pairwise genetic correlation (r_g) between education and employment traits with HCV-negative HCC on inclusion and exclusion of alcohol-associated loci.

Supplementary Table 1. SNPs associated with Alcohol Consumption.

Supplementary Table 2. Pairwise comparisons and LDSR results of HCV-negative HCC.

Supplementary Table 3. The shared genetic correlations of HCC on the inclusion/exclusion of genomic regions related to alcohol genetic behaviors.

Supplementary tables are available in a separate file.

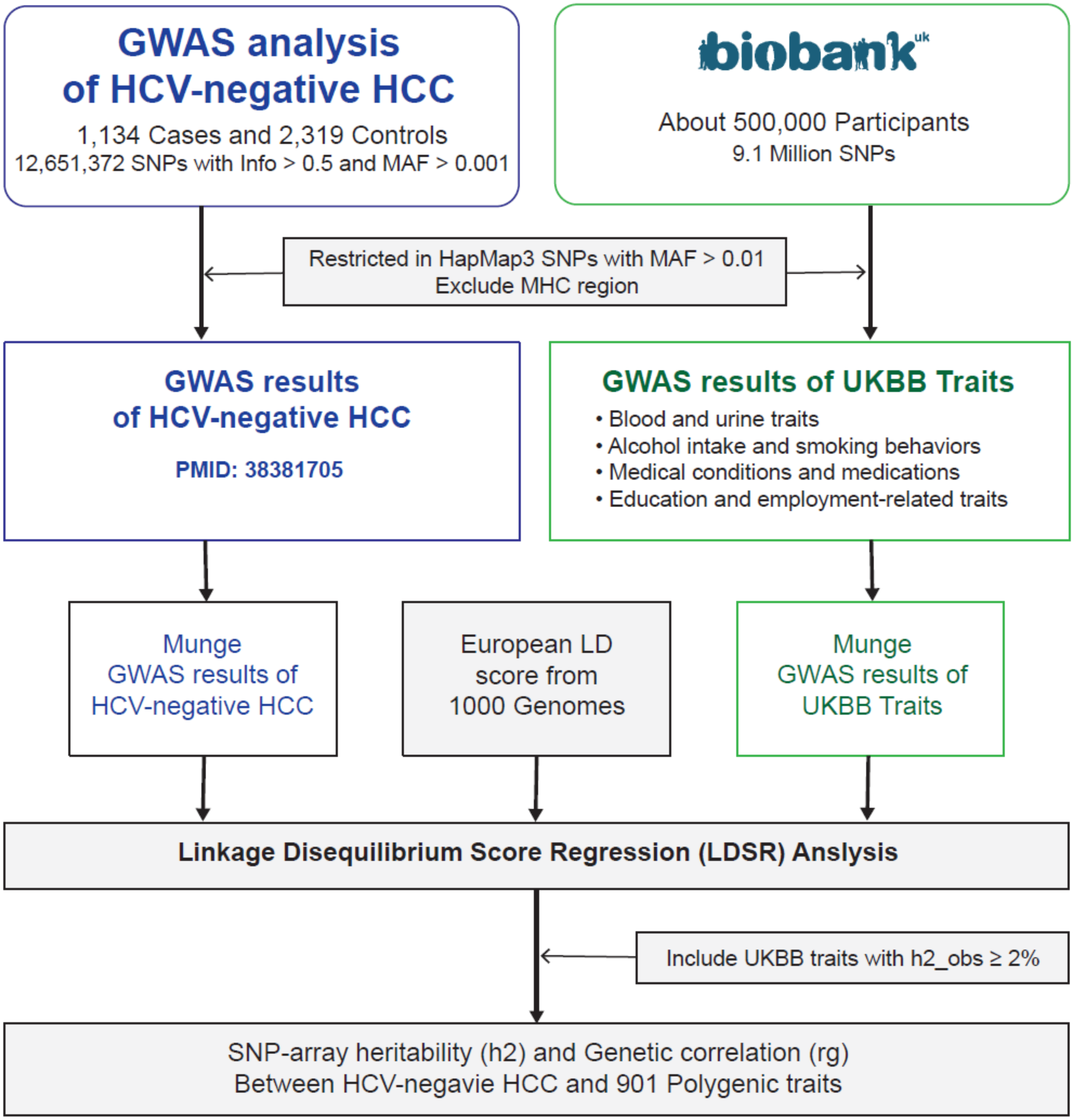

Supplementary Figure 1. Study design schematic for identifying the shared genetic etiology underlying HCC.

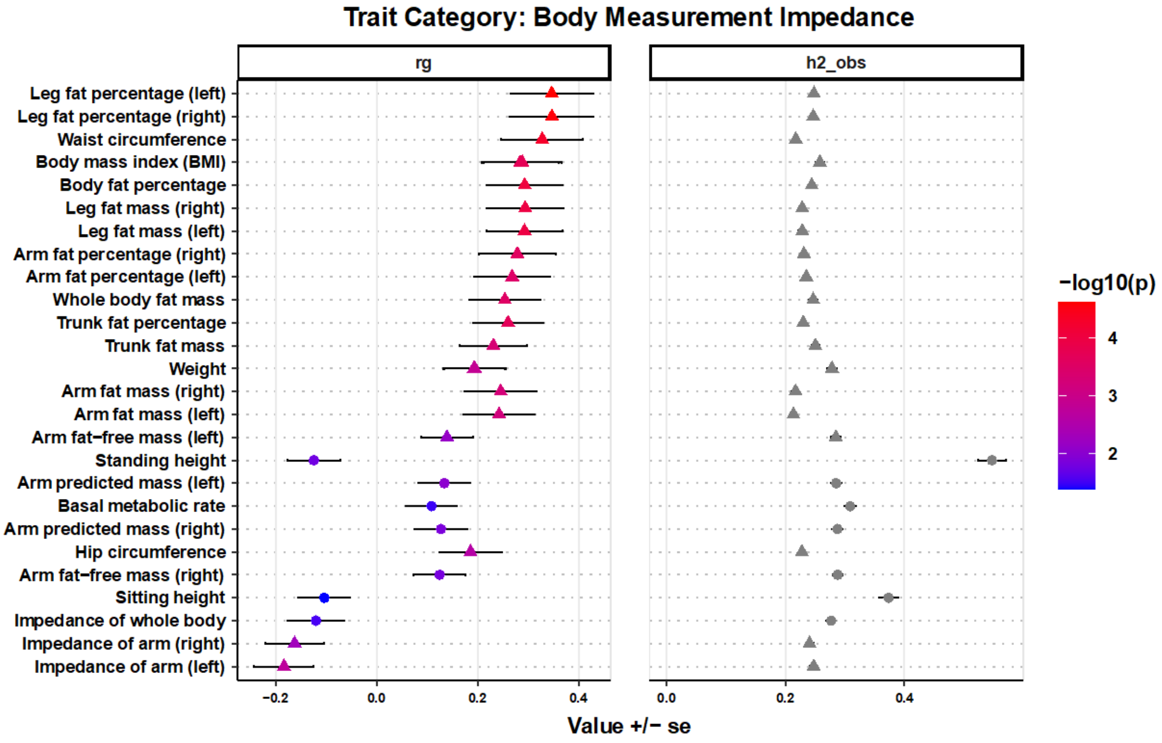

Supplementary Figure 2. Genetic correlation (r_g) and observed scale heritability (h2_obs) between body measurement impedance with HCV-negative HCC. The circle indicates nominal significance ($p \leq 5\times10^{-2}$), while the triangle indicates FDR-adjusted significance ($p \leq 8.02\times10^{-3}$).

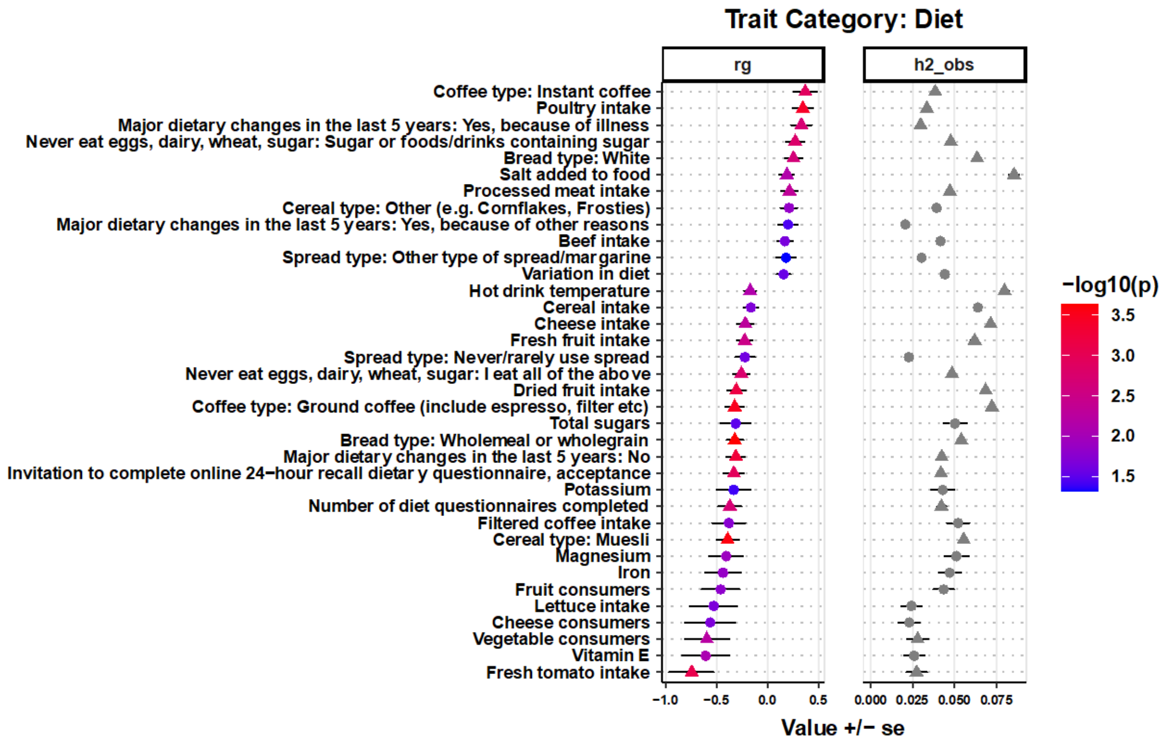

Supplementary Figure 3. Genetic correlation (r_g) and observed scale heritability (h2_obs) between diet-related traits with HCV-negative HCC. The circle indicates nominal significance ($p \leq 5\times10^{-2}$), while the triangle indicates FDR-adjusted significance ($p \leq 8.02\times10^{-3}$).

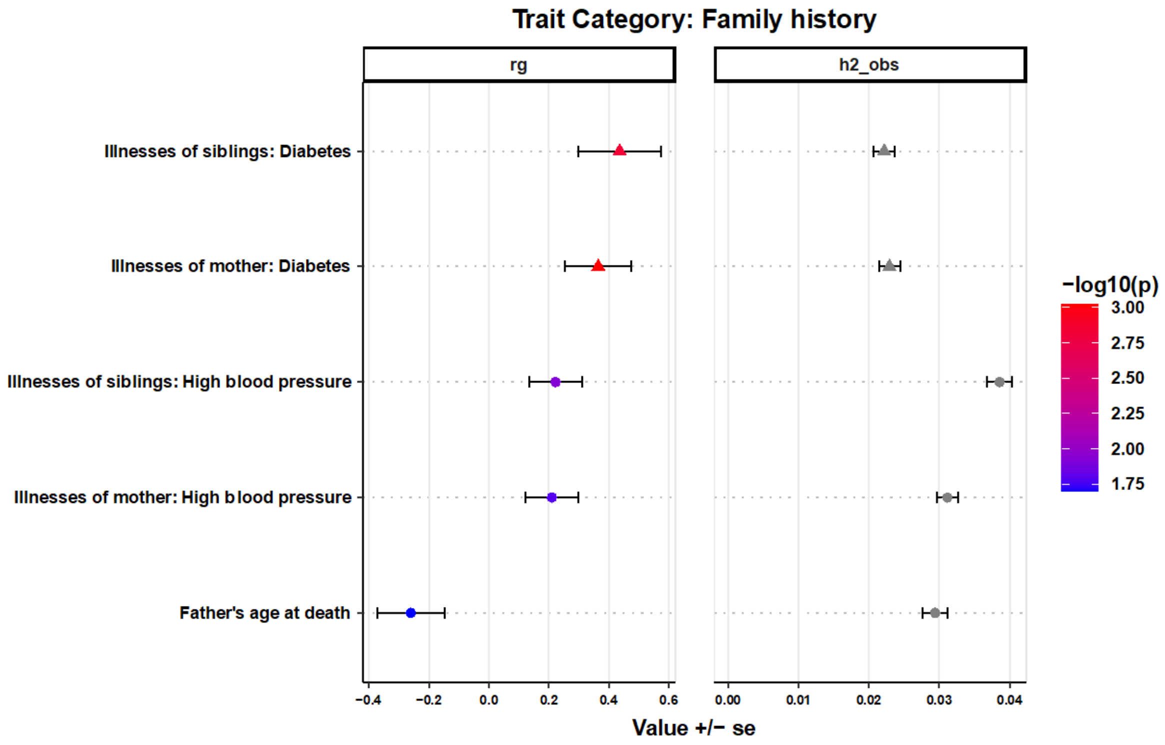

Supplementary Figure 4. Genetic correlation (r_g) and observed scale heritability (h2_obs) between family history traits with HCV-negative HCC. The circle indicates nominal significance ($p \leq 5\times10^{-2}$), while the triangle indicates FDR-adjusted significance ($p \leq 8.02\times10^{-3}$).

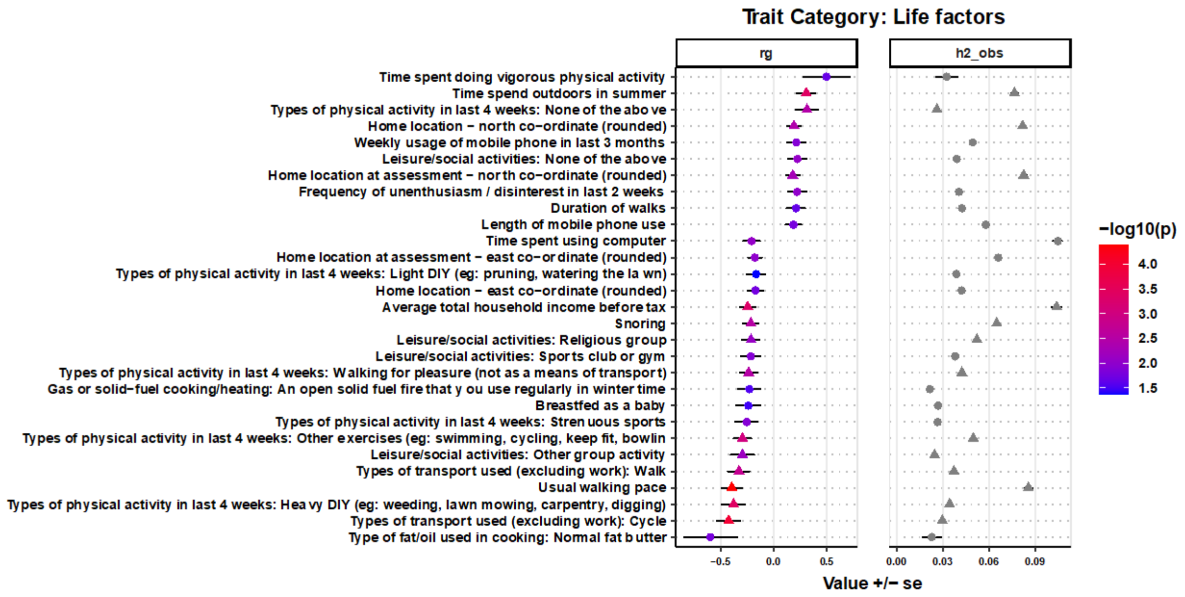

Supplementary Figure 5. Genetic correlation (r_g) and observed scale heritability (h2_obs) between traits related to various life factors with HCV-negative HCC. The circle indicates nominal significance ($p \leq 5\times10^{-2}$), while the triangle indicates FDR-adjusted significance ($p \leq 8.02\times10^{-3}$).

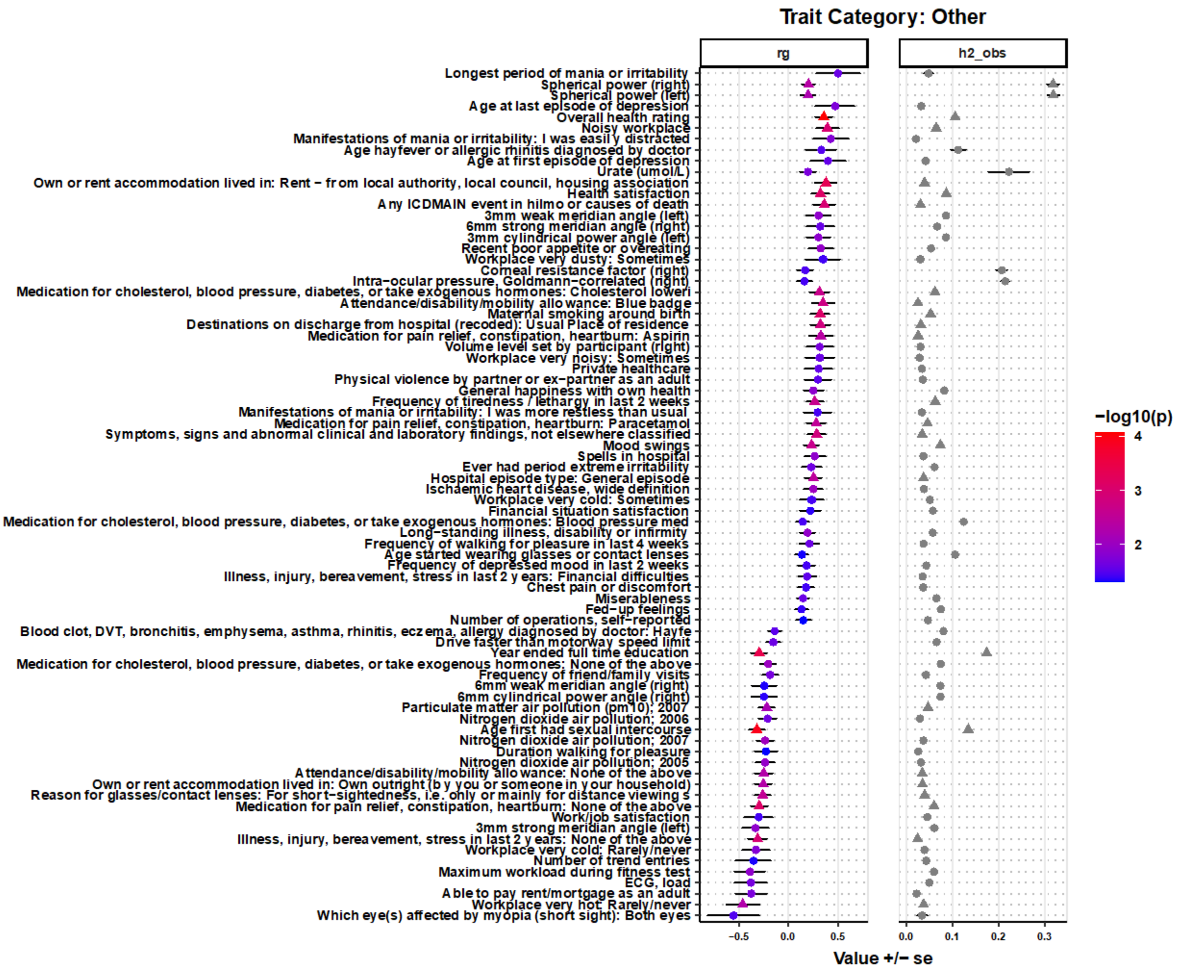

Supplementary Figure 6. Genetic correlation (r_g) and observed scale heritability (h2_obs) of other traits with HCV-negative HCC. The circle indicates nominal significance ($p \leq 5\times10^{-2}$), while the triangle indicates FDR-adjusted significance ($p \leq 8.02\times10^{-3}$).

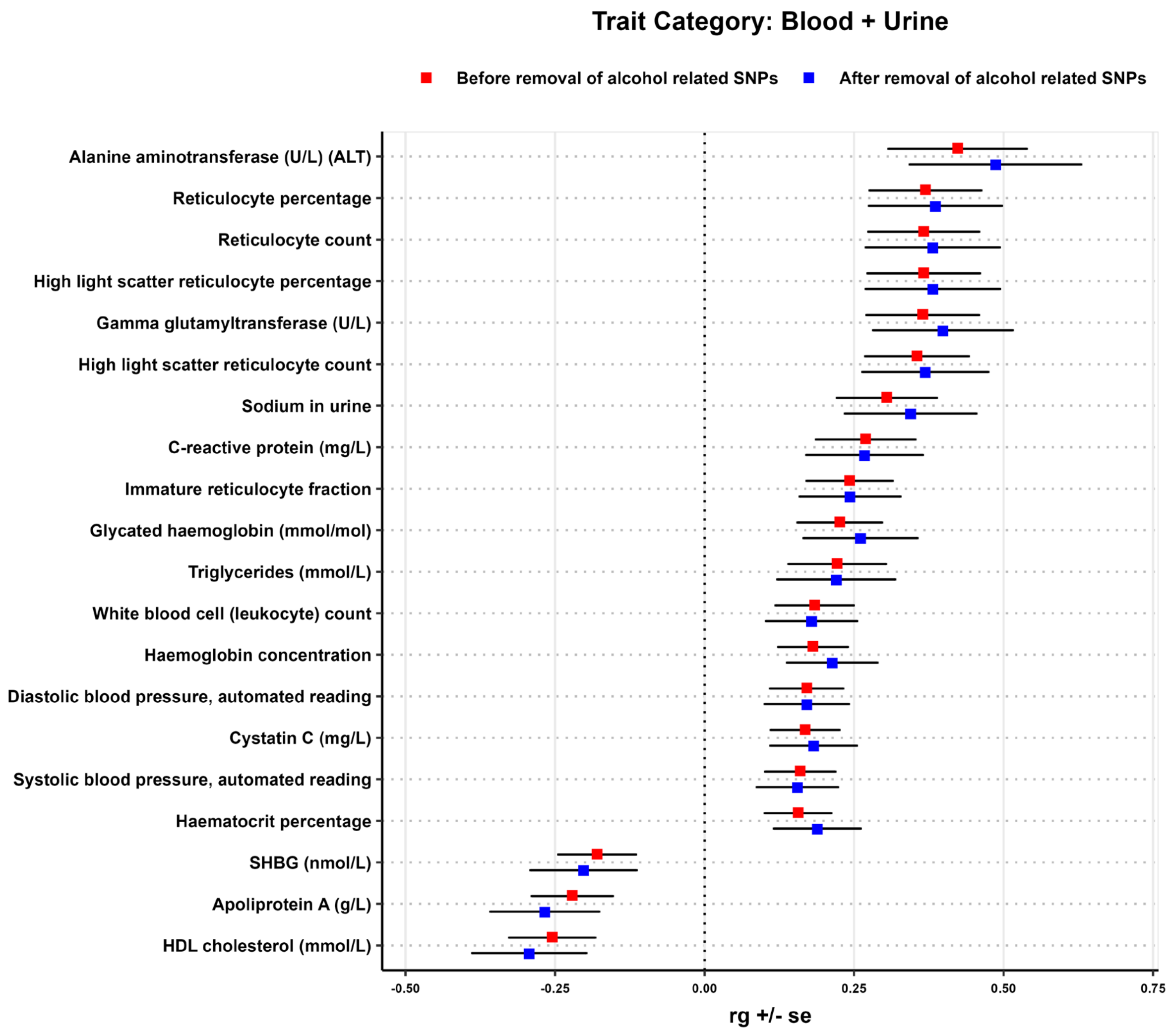

Supplementary Figure 7. Pairwise genetic correlation (r_g) between blood and urine traits with HCV-negative HCC on inclusion and exclusion of alcohol-associated loci.

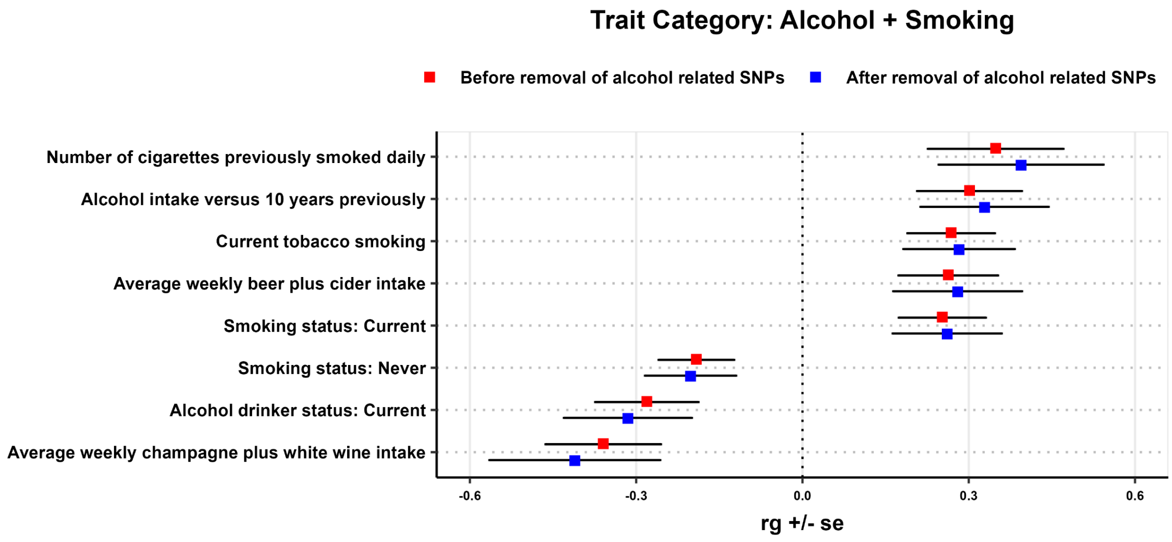

Supplementary Figure 8. Pairwise genetic correlation (r_g) between alcohol and smoking traits with HCV-negative HCC on inclusion and exclusion of alcohol-associated loci.

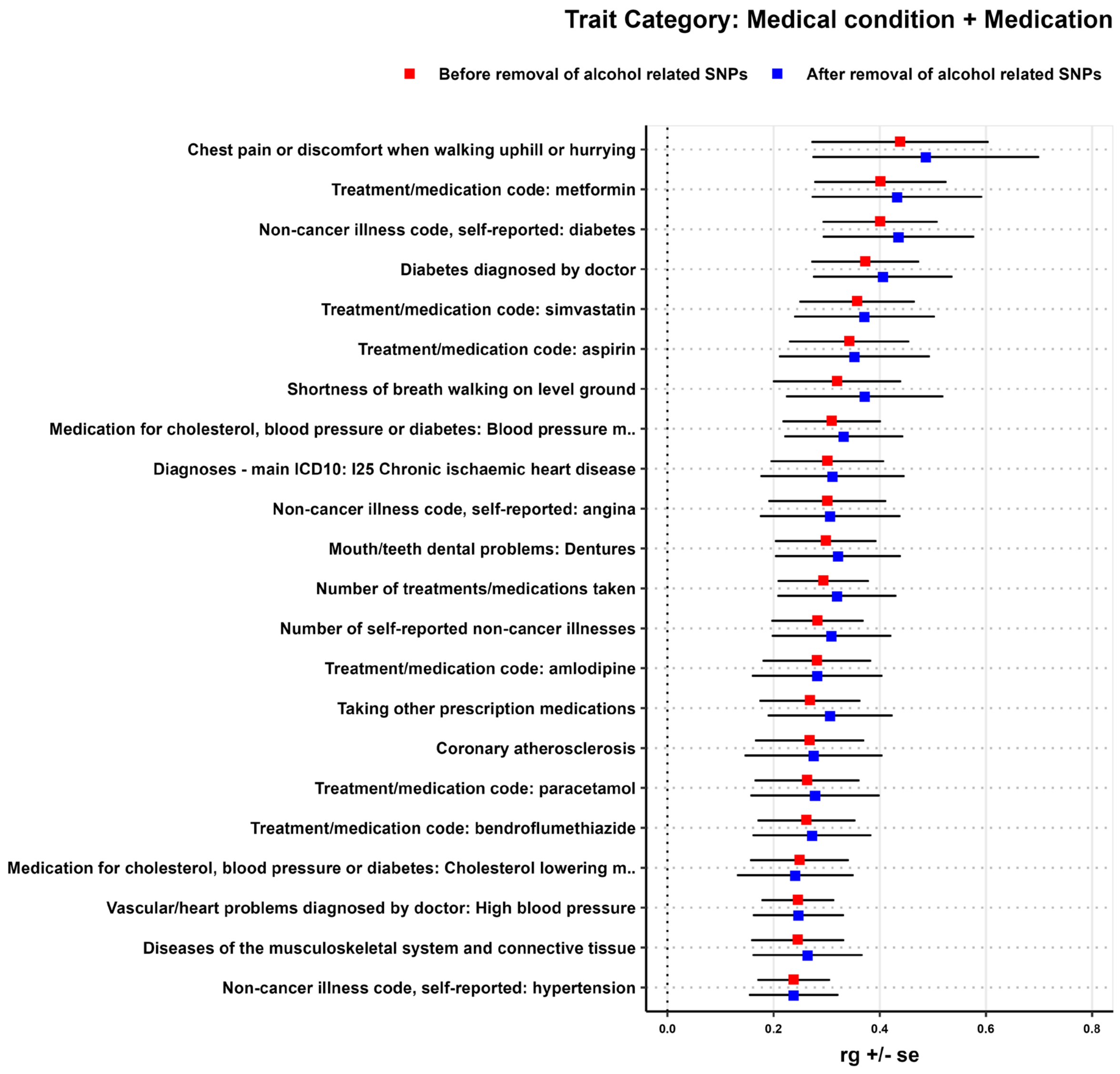

Supplementary Figure 9. Pairwise genetic correlation (r_g) between medical conditions and medication traits with HCV-negative HCC on inclusion and exclusion of alcohol-associated loci.

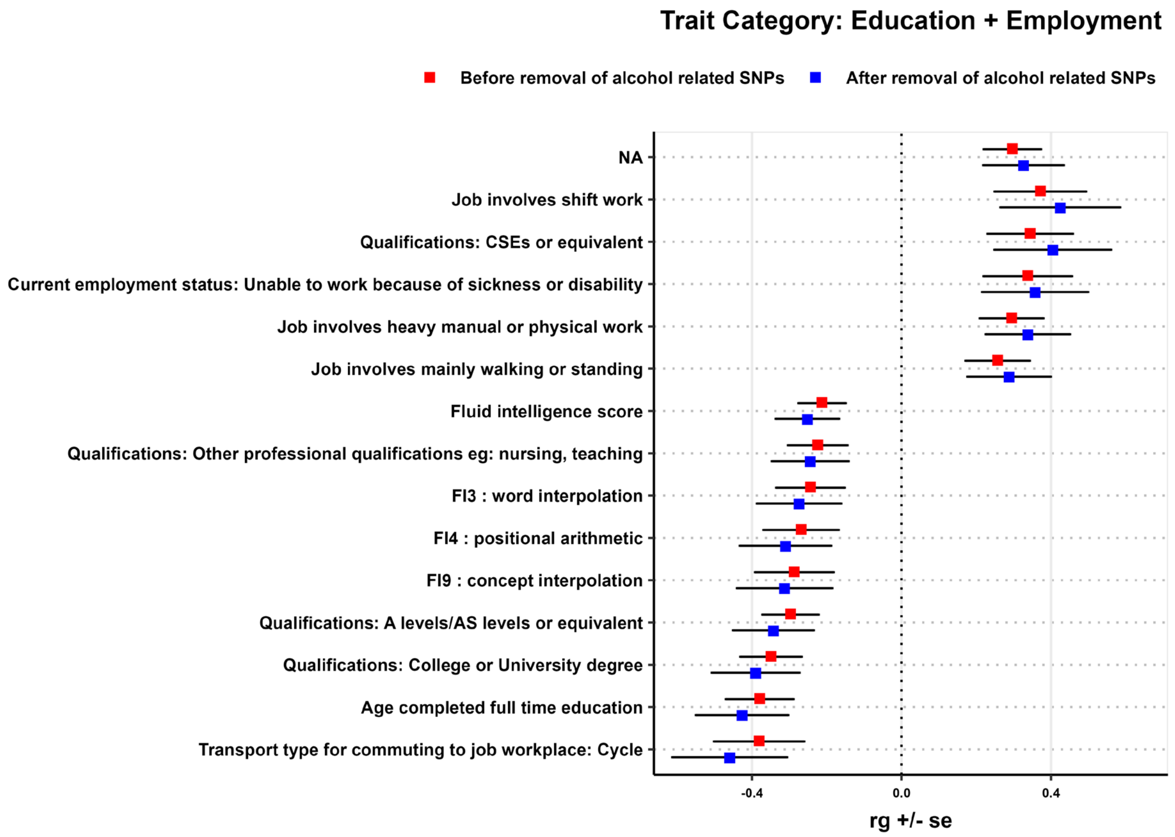

Supplementary Figure 10. Pairwise genetic correlation (r_g) between education and employment traits with HCV-negative HCC on inclusion and exclusion of alcohol-associated loci.
